# Mode of Testing the Translucency of Hydrocele

**Published:** 1856-12

**Authors:** 


### *Mode of Testing the Translucency of Hydrocele.*

As ordinarily employed, by placing a candle on one side of the tumor, and excluding the passage of the light laterally by means of the hand, it is, at best, a clumsy proceeding, and liable to errors. I have found the stethoscope much more useful, as a means of excluding the diffused light, and by applying the eye to its expanded bell-shaped portion—the ear-piece being firmly placed upon the scrotum, held in a tense condition—we can even map out the state of the parts with tolerable accuracy, if the contained fluid be of ordinary character, and detect the condition of the testicle by the opacity it produces, especially when it occupies any unusual locality, as the front or sides of the scrotum, or is adherent from inflammation after previous tappings. We can employ either a lighted candle or bright sunlight, as our best means of obtaining the requisite illumination; but even in diffused daylight I have succeeded very well in the manner I mention.—Dr. W. FRAZER, in *Dublin Hospital Gazette.*

